# Feasibility and Effectiveness of Personalized Home‐Based Neurostimulation for Teachers Experiencing Work‐Related Rumination

**DOI:** 10.1002/brb3.71264

**Published:** 2026-02-24

**Authors:** Delia Ciobotaru, Vu Nguyen, Alejandro Pérez, Zoe E. Clothier, Ines R. Violante, Mark Cropley, Roi Cohen Kadosh

**Affiliations:** ^1^ School of Psychology Faculty of Health and Medical Sciences University of Surrey Guildford UK; ^2^ Amazon Adelaide Australia; ^3^ Dpto. Psicología Cognitiva, Social y Organizacional Universidad De La Laguna Tenerife Spain; ^4^ School of Biomedical Engineering and Imaging Sciences Faculty of Life Sciences and Medicine, King's College London London UK; ^5^ Cognite Neurotechnology Ltd Oxford UK

**Keywords:** home‐based, neurostimulation, personalization, rumination, teachers

## Abstract

**Introduction:**

Work‐related rumination is associated with poor mental and physical health. This study aimed to develop and evaluate a personalized Bayesian optimization (pBO) algorithm, designed to tailor transcranial alternating current stimulation (tACS) parameters for UK schoolteachers experiencing elevated work‐related rumination.

**Methods:**

The algorithm adjusted tACS parameters based on individual head circumference and rumination levels. During the development phase, 80 burn‐in and 319 personalized home‐based sessions were conducted with 67 schoolteachers to model parameter–outcome relationships. In a preregistered, double‐blind, within‐participant follow‐up study, 38 schoolteachers received both personalized and sham stimulation sessions in a counterbalanced order. Measures included work‐related rumination, daytime sleepiness, actigraphy‐based sleep fragmentation and efficiency, and side‐effect ratings.

**Results:**

In the algorithm development phase, higher‐amplitude stimulation was associated with reduced sleep fragmentation. In the follow‐up study, both personalized and sham stimulation reduced rumination, but no significant differences were observed between conditions. Higher amplitudes were associated with greater reductions in daytime sleepiness. Post‐stimulation changes in sleepiness and ruminations were associated only at higher amplitudes in the personalized condition and were not observed following sham stimulation. Side‐effect severity did not differ significantly between conditions.

**Conclusions:**

Home‐based neurostimulation interventions are feasible and well tolerated. While personalized stimulation did not outperform sham, the findings identify current amplitude as a key factor influencing daytime sleepiness and highlight the need to further refine personalization algorithms and stimulation strategies to optimize effectiveness.

## 1 Introduction

Work‐related rumination refers to the persistent and repetitive contemplation of work‐related issues outside of working hours (Querstret and Cropley 2012). This process can take different forms. Some people engage in problem‐solving pondering, where they focus on finding solutions to work challenges. Others experience more negative or unproductive thoughts, known as affective rumination, which yield negative emotional responses such as stress, anxiety, and frustration (Kinnunen et al. 2017), as well as lower job satisfaction and engagement (Mullen et al. 2020). At the level of clinical morbidity, affective work‐related rumination is robustly associated with higher risk of non‐psychotic psychiatric morbidity including anxiety and depression (Vandevala et al. 2017). Regarding symptom burden and functional impairment, studies indicate greater fatigue (Firoozabadi et al. 2018), increased insomnia and sleep disturbance symptoms (Kinnunen et al. 2017; Melo et al. 2021; Pauli et al. 2023), and lower executive functioning skills (Cropley and Collis 2020). For physiological biomarkers, emerging evidence associates work‐related rumination with increased cortisol awakening response (Cropley et al. 2015) and lower heart rate variability (Cropley et al. 2017).

Knowledge workers are especially vulnerable to persistent negative work‐related thinking, with evidence across public‐service roles such as social workers (Eggli et al. 2021), aid workers (Wen et al. 2021), and police officers (Juczyński and Ogińska‐Bulik 2022). Within this group, schoolteachers are a salient and highly studied case, since after‐hours planning and marking, together with classroom demands and student behavior, sustain work‐related rumination beyond the school day (Cropley and Millward Purvis 2003; Türktorun et al. 2020; Montgomery and Rupp 2005). These dynamics can also reduce the quality and consistency of instruction (Harrison et al. 2023; Klusmann et al. 2008), linking teacher wellbeing to student outcomes and, ultimately, societal benefit.

Given the burden in teaching, interest has turned to interventions that can be deployed alongside work schedules. A small number of studies have examined non‐invasive neurostimulation as a potential solution; however, efforts to reduce rumination with transcranial direct current stimulation (tDCS) have yielded heterogeneous effects, likely reflecting one‐size‐fits‐all targeting, methodological caveats, and protocol variability (Hoebeke et al. 2021). This variability, together with converging evidence that efficacy improves when stimulation entrains endogenous oscillations at circuit‐relevant frequencies, points to the therapeutic potential of frequency‐specific protocols (Yener and Başar 2013). On this basis, we focus here on transcranial alternating current stimulation (tACS) (Antal et al. 2017; Antal et al. 2022), an efficient, low‐cost, non‐invasive neurostimulation method, which is unique in its capacity to modulate intra‐ and interarea neural oscillations in a frequency‐specific manner (Herrmann et al. 2013). tACS has already shown promising neural, cognitive, and emotional effects in healthy participants (Antonenko et al. 2016; van Driel et al. 2015) and a limited range of clinical populations (Ahn et al. 2019; Alexander et al. 2019; Dallmer‐Zerbe et al. 2020). For example, preliminary evidence suggests that theta‐range tACS, the most commonly employed frequency band in tACS research (Grover et al. 2023), may enhance cognitive control and working memory (van Driel et al. 2015; Jaušovec and Jaušovec 2014; Yu et al. 2020). Such enhancements could potentially benefit individuals prone to rumination by improving their ability to inhibit or shift attention away from negative thoughts. Reviews have also stressed the potential of tACS to modulate disturbed oscillations in neuropsychiatric disorders (Pan et al. 2023; Elyamany et al. 2021; Strüber and Herrmann 2020), thus highlighting the timeliness of this line of research and the importance of exploring the effectiveness of tACS on transdiagnostic measures relevant to disorders stemming from and resulting in emotional vulnerability.

Traditionally, researchers have predominately chosen stimulation parameters based on prior experimental designs rather than personalized neurobiological insights. This approach overlooks individual differences and therefore does not maximize performance outcomes, as not everyone responds equally to the same stimulation parameters (Krause and Cohen Kadosh 2014). In contrast, a bottom‐up approach allows the exploration of different neurostimulation protocols, potentially uncovering more promising parameter combinations that have not yet been examined. Consequently, researchers are moving away from one‐size‐fits‐all interventions towards treatments tailored to individuals’ characteristics, with artificial intelligence and machine learning providing a potential method for personalization.

To operationalize this shift, we implemented a personalized variant of Bayesian optimization (BO), a machine learning technique that seeks the global optimum of a black‐box function by creating a surrogate model and using it to select the next optimal evaluation (Strüber and Herrmann 2020). BO effectively explores large experimental spaces, identifying the best parameter combinations for treatment. While previous BO approaches (Lorenz et al. 2019; Tervo et al. 2020) incorporated personalization by testing different stimulation parameters within the same participant, personalized BO (pBO) improves upon this by accumulating knowledge across participants to propose a single set of parameters for each new participant or session. This iterative learning process enhances the algorithm's performance as more data becomes available, allowing it to more efficiently explore a reduced number of stimulation parameter combinations (van Bueren et al. 2021).

Evidence for algorithmically personalized neurostimulation is promising but nascent: to our knowledge, one recent home‐based study reported that a pBO pipeline tailoring transcranial random noise stimulation (tRNS) parameters to baseline ability and head circumference improved sustained attention, with validation spanning in‐silico modeling and a double‐blind, sham‐controlled design (Cohen Kadosh et al. 2025). While encouraging, this leaves open questions about durability, boundary conditions, and generalization to other outcomes. These gaps motivate replications as well as extensions to new targets and populations, including our present examination of pBO for reducing affective work‐related rumination in teachers.

Building on this, we developed and evaluated a pBO algorithm to personalize tACS parameters with the aim of reducing affective work‐related rumination in teachers. In Study 1, we developed the pBO algorithm suggesting the optimal tACS amplitude and frequency based on participants’ head circumference and baseline affective rumination scores. These two variables were chosen for their relevance to stimulation effectiveness, as follows. Head circumference served as a proxy for estimating differences in the electric field strengths induced by transcranial electrical stimulation (tES), as larger head circumference was found to be associated with lower electric field strengths across electrode montages (Antonenko et al. 2021). Consistent with this, Supplementary Figure 1 (1 mA tACS, F3–CZ) shows that a larger head circumference yields a weaker and less extensive E‐field across the targeted regions. Moreover, affective work‐related rumination was considered a proxy for disrupted brain oscillations associated with repetitive thought patterns, as both adaptive and maladaptive rumination have been directly linked to alterations in the spatiotemporal complexity of these oscillations in major depressive patients (Wang et al. 2022).

In Study 2, we evaluated the effectiveness of the personalized home‐based intervention in a preregistered, double‐blind, sham‐controlled study. We hypothesized that personalized stimulation would lead to greater reductions than sham in affective work‐related rumination, with mental health‐related traits, such as depression and anxiety, potentially moderating this relationship. Secondary outcomes included changes in self‐reported state sleepiness as well as in physiological measures of sleep quality.

### 1.1 Preregistration

Study 1 focused on algorithm development and was therefore not preregistered. Study 2's design and analyses were preregistered on AsPredicted (ID 158274: “reducing work‑related rumination through personalized neurostimulation”): https://aspredicted.org/ppr4‐jm5z.pdf.

### 1.2 Data Analysis

Descriptive statistics, sequential Bayesian analysis, *t*‐tests, and ANOVA were performed in JASP (v0.95.1). Data visualization and linear mixed‐effects modeling were performed in R 4.5.1 via RStudio 2025.05.1 + 513.

## 2 Study 1: Algorithm Development

### 2.1 Material and Methods

#### 2.1.1 Participants

Participants were 67 UK‐based schoolteachers (40 females, 27 males) with high levels of affective work‐related rumination, having a score of at least 15 out of 25 on the affective scale of the Work‐rRlated Rumination Questionnaire (Cropley et al. 2012). The participants’ ages ranged from 25 to 60 years (*M* = 41.2, *SD* = 9.11). Participants were excluded if they had been in treatment for psychological or psychiatric problems in the past two years; had a history of neurological injuries; were using psychoactive medication; consumed more than 15 units of alcohol per week on average; used recreational drugs in the past month; had a history of epilepsy or had a first‐degree relative with a history of epilepsy; or were pregnant. Participants were recruited in the online environment on social media platforms (Facebook, Instagram, LinkedIn, Twitter) and via email campaigns from Schoolzone UK and the Association of School and College Leaders. Teachers received £15 reimbursement per study session, with an additional £15 to compensate for time spent on equipment collection and return.

#### 2.1.2 Materials

##### 2.1.2.1 Questionnaires

The Cognitive Failures Questionnaire (CFQ) (Broadbent et al. 1982) comprises 25 items assessing everyday cognitive lapses, rated on a 5‐point scale (1 = never, 5 = very often; α = 0.90 (Cropley et al. 2012)). The Generalized Anxiety Disorder 7‐item (GAD‐7) (Rast et al. 2009) assesses trait anxiety, with items rated on a 4‐point scale (0 = not at all, 3 = nearly every day; α = 0.87 (Spitzer et al. 2006)). The Patient Health Questionnaire 8‐item (PHQ‐8) (Kroenke and Spitzer 2002) measures trait depression, adapted from the PHQ‐9 by omitting the item on suicidal ideation. The PHQ‐8 items are rated on a 4‐point scale (0 = not at all, 3 = nearly every day; α = 0.80–0.96 (Kroenke et al. 2010)). The Pittsburgh Sleep Quality Index (PSQI) (Buysse et al. 1989) comprises 19 items forming seven components that yield a global score (0–21; α = 0.83 (Buysse et al. 1989)), with higher scores indicating poorer sleep quality. The Work‐Related Rumination Questionnaire (WRRQ) (Cropley et al. 2012) affective subscale includes 5 items (1 = very seldom/never, 5 = very often/always; α = 0.90 (Querstret and Cropley 2012)). The Stanford Sleepiness Scale (SSS) (Hoddes et al. 1973) is a single‐item measure assessing perceived daytime sleepiness on a seven‐point scale (1 = feeling active and vital, 7 = almost in reverie, sleep onset soon).

##### 2.1.2.2 Equipment

Participants were provided with an internet‐enabled tablet and the CE‐marked Starstim‐Home tES Device (Neuroelectrics, Barcelona, Spain) for their home‐based neurostimulation sessions. Sleep was monitored using MotionWatch 8 CE‐marked actigraphy watches (CamNtech, Cambridge, UK). Sleep and movement data were collected in 60‐s epochs. The output, processed through the accompanying software (MotionWare 1.3.33, CamNtech), was used to analyse sleep efficiency and sleep fragmentation.

#### 2.1.3 Design and Procedure

For BO with noise, as in the case of human‐based research, previous studies have set the number of evaluations to 25–50 per dimension (van Bueren et al. 2021; Letham et al. 2019). To accommodate the higher variability of a home‐based study, we set 100 evaluations per continuous dimension, requiring 400 data points to account for four continuous dimensions: current amplitude, stimulation frequency, baseline affective work‐related rumination, and head circumference. Each participant completed 5–7 sessions, yielding 399 sessions. The stimulation included 30 s ramp‐in/ramp‐out phases and 20 min of tACS (1–100 Hz, 1 Hz steps; 0.1–1.6 mA peak‐to‐peak, 0.1 mA steps). The electrodes were placed over the left dorsolateral prefrontal cortex (LDPFC) and the midline central region of the head, as motivated by previous fMRI studies attesting the role of the LDPFC in rumination (Cooney et al. 2010; Kowalski et al. 2019).

A burn‐in phase of 80 random tACS parameters was applied to the first 16 participants to initiate the pBO procedure. After the burn‐in phase, the pBO procedure was initiated before each session and updated with the rumination outcomes afterward. This was done in an iterative process, with the algorithm's estimate of the optimal stimulation parameter, at any given head circumference and baseline level of work‐related rumination, becoming more accurate as more participants were tested. At each run of the pBO algorithm, all previously collected data were used, including data collected in the burn‐in phase, and the surrogate Gaussian process (GP) was refitted to model the data.

Participants received an actigraphy watch to wear nightly from one night before the first stimulation session until the night of the final session, pressing the event marker at bedtime and waking. They also measured their head circumference using a provided tape measure, and completed trait questionnaires (CFQ, GAD‐7, PHQ‐8, and PSQI) via Qualtrics. Stimulation sessions were scheduled Monday‐Thursday on teaching days. On the morning of each session, participants completed the SSS and the state version of the WRRQ affective scale. The experimenter used the affective WRRQ and head circumference inputs to generate the appropriate stimulation parameters using the pBO algorithm. In the evening, participants repeated the SSS and affective WRRQ to provide baseline data for the session and followed on‐screen setup instructions via their tablet. The experimenter approved the electrode placements via a video call before the first stimulation session. For subsequent sessions, participants could either request additional video call guidance or follow the tablet instructions independently. During stimulation, participants engaged in a relaxing, seated activity and later reported it in a free‐text response box. Following each session, they re‐completed the SSS and affective WRRQ and recorded any adverse events.

### 2.2 Results

#### 2.2.1 Algorithm Development

Across 399 home‐based sessions, the optimization routine explored the amplitude–frequency space rather than fixating on a narrow set of settings. The frequency and progression of sampling different parameter combinations across the 399 sessions are illustrated in Supplementary Figure 2. The most frequently sampled combination was an amplitude of 0.7 mA and a frequency of 72.3 Hz (*n* = 132), followed by an amplitude of 1.3 mA at a frequency of 1.0 Hz (*n* = 10).

#### 2.2.2 Descriptive Statistics

**FIGURE 1:**
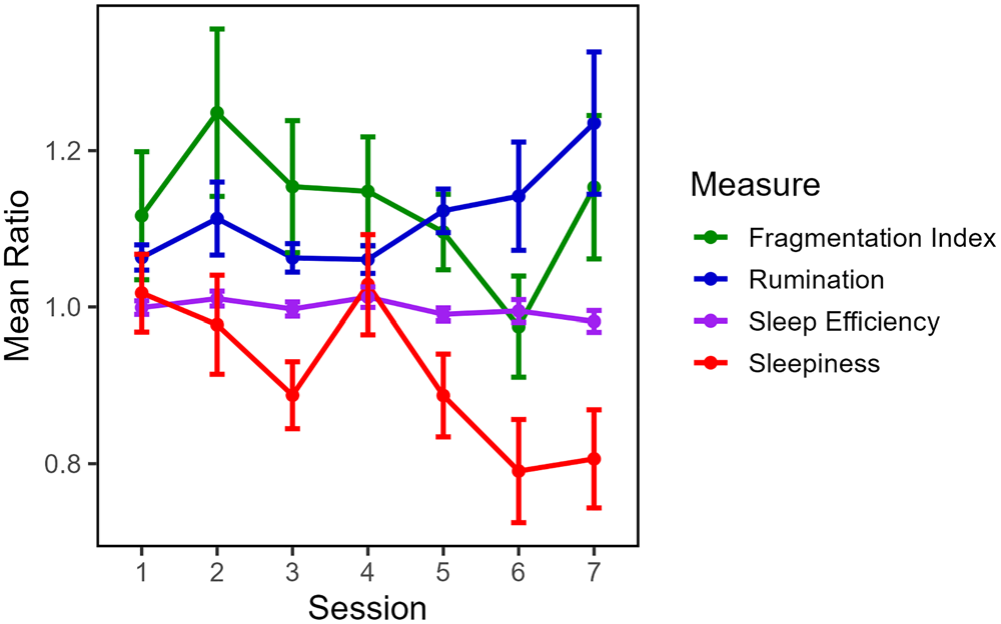
Changes in state measure ratios across sessions. Note: ratios are defined as pre‐/post‐stimulation scores (values > 1 indicate reductions from pre‐stimulation; larger ratios = larger reductions) points show session means (sessions 1–5: *n* = 67; sessions 6–7: *n* = 31). Error bars show 95% confidence intervals.

Descriptive statistics for the trait questionnaires are summarized in Supplementary Table 1. Session‐wise trajectories for state measures are shown in Figure 1, using a pre/post ratio (values > 1 indicate reductions from pre‐ to post‐stimulation). For sleep, analyses focused on two key actigraphy metrics: sleep efficiency, which measures the proportion of time spent asleep in relation to the total time in bed, and the fragmentation index, which combines periods of movement and very short episodes of stillness, reflecting the amount of restlessness or interruptions during sleep.

#### 2.2.3 Predicted Affective Rumination Reduction

**FIGURE 2:**
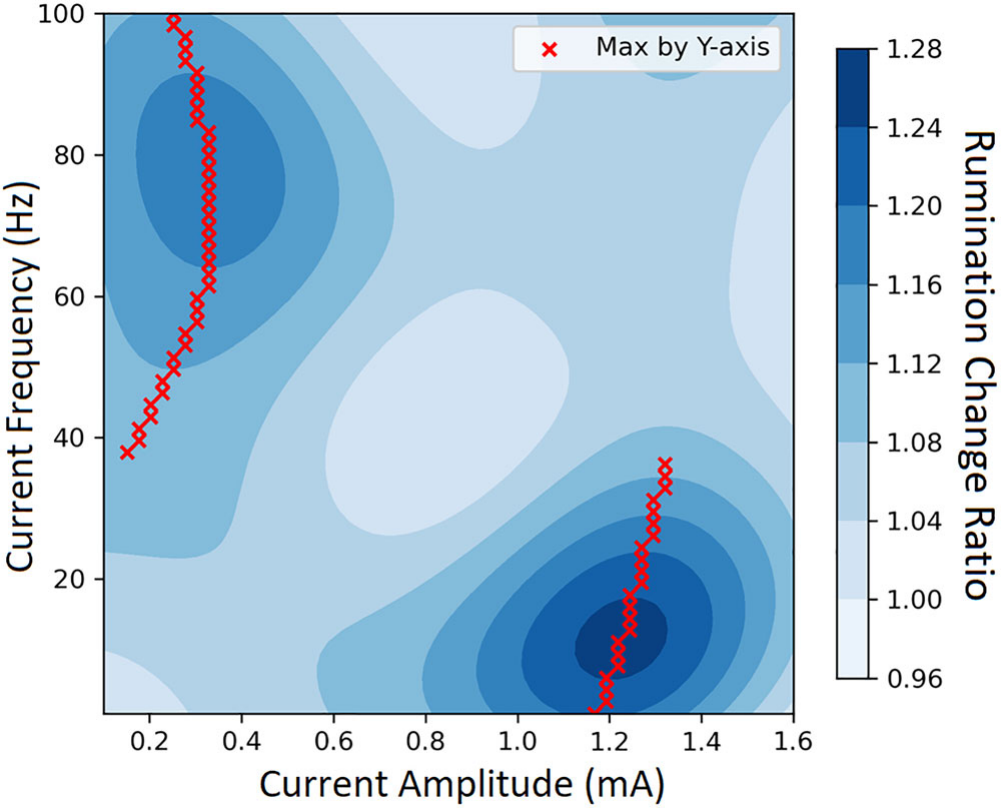
Predicted reduction in affective rumination by tACS amplitude–frequency. Note: heatmap shows the model‐estimated change in rumination, defined as pre‐/post‐stimulation levels of rumination (values > 1 denote a reduction; larger values indicate greater reduction), according to combinations of current amplitude and frequency across all head circumferences and baseline rumination levels. Higher current amplitudes combined with lower frequencies are predicted to generate the greatest reductions in rumination.

**FIGURE 3:**
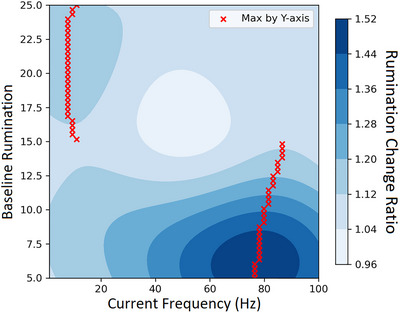
Predicted reduction in affective rumination by tACS frequency and baseline rumination. Note: heatmap shows the model‐estimated change in rumination, defined as pre‐/post‐stimulation levels of rumination (values > 1 denote a reduction; larger values indicate greater reduction), according to combinations of current frequency and baseline rumination across all head circumferences and current amplitudes. Lower frequencies seem to be most effective in reducing rumination for individuals experiencing higher levels of baseline rumination.

**FIGURE 4:**
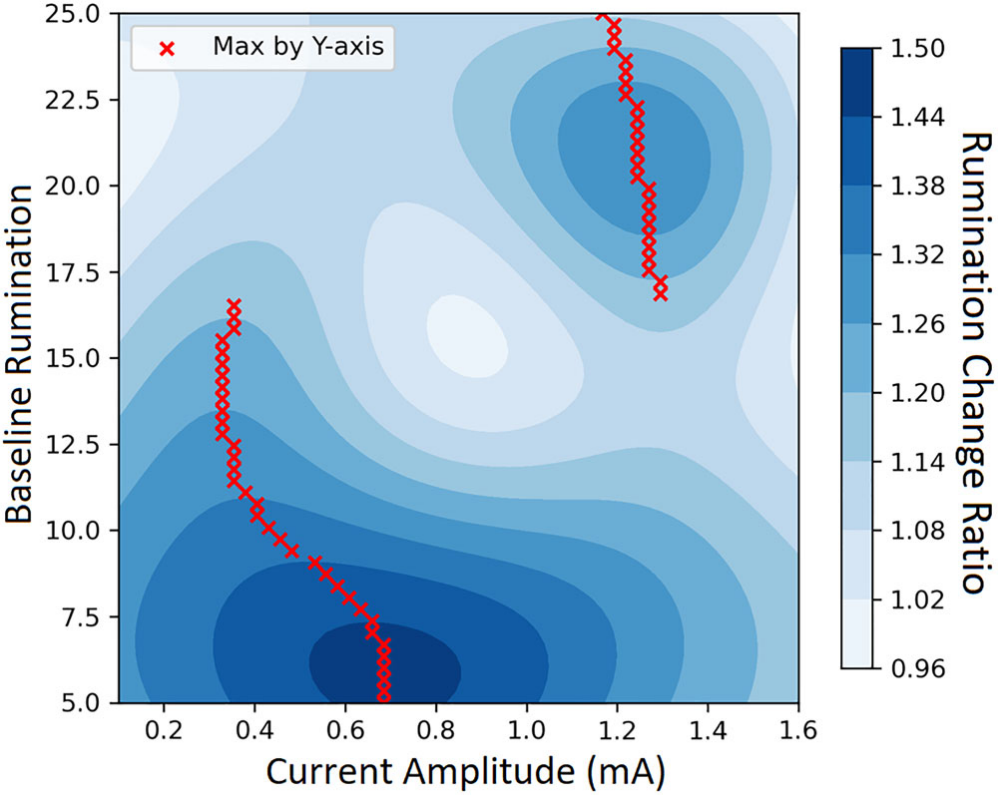
Predicted reduction in affective rumination by tACS amplitude and baseline rumination. Note: heatmap shows the model‐estimated change in rumination, defined as pre‐/post‐stimulation levels of rumination (values > 1 denote a reduction; larger values indicate greater reduction), according to combinations of current amplitude and baseline rumination across all head circumferences and current frequencies. Higher amplitudes seem to be most effective in reducing rumination for individuals experiencing higher levels of baseline rumination.

We describe the performance and plot the results based on the combination of different dimensions. The optimal combinations to achieve greater reductions in rumination across head circumferences and baseline rumination appear to be higher amplitude (around 1.0–1.4 mA) and lower frequency (approximately up to 25 Hz), followed by lower amplitude (around 0.2–0.4 mA) and higher frequency (around 65–90 Hz). In contrast, a combination of moderate amplitude (0.6–1.0 mA) and frequency (around 25 to 60 Hz) might be detrimental, leading to increased rumination (see Figure 2). Across head circumferences and current amplitudes, individuals with higher baseline rumination respond more favourably to lower‐frequency stimulation, up to around 25 Hz (see Figure 3). Conversely, when considering head circumferences and current frequencies, individuals with higher baseline rumination experience greater rumination reductions with higher‐amplitude stimulation ranging from 1.0 mA to 1.4 mA (see Figure 4).

#### 2.2.4 Effects of Amplitude on Fragmentation Index

To examine same‐night sleep quality as per the actigraphy metrics, a linear mixed‐effects model (estimated using REML and nloptwrap optimizer) was conducted to examine the effects of current amplitude and fragmentation index from the night before (pre‐fragmentation index) on the post‐stimulation fragmentation index (formula: fragmentation index ∼ current amplitude + pre‐fragmentation index). The model included session, the session weekday and participants as random effects (formula: ∼1|session, ∼1|weekday, ∼1|participant). The model's explanatory power related to the fixed effects alone (marginal R^2^) was 0.15. Within this model, current amplitude had a significant main effect, with higher current amplitude leading to a greater reduction in fragmentation index (*β* = –0.11, 95% CI[–0.20, –0.02], *t*(329) = –2.36, *p* = 0.02). The main effect of pre‐fragmentation index was also statistically significant, with a greater pre‐fragmentation index predicting a greater fragmentation index following stimulation (*β* = 0.30, 95% CI[0.19, 0.41], *t*(329) = 5.51, *p* < 0.001). Therefore, after adjusting for previous‐night sleep fragmentation, higher current amplitude predicted lower same‐night sleep fragmentation.

## 3 Study 2: Algorithm Effectiveness

### 3.1 Material and Methods

#### 3.1.1 Participants

Participants had a similar profile to those in Study 1, consisting of 18 females and 20 males, with ages ranging from 24 to 56 years (*M* = 42.4, *SD* = 8.5). Additionally, individuals who had previously participated in Study 1 were deemed ineligible for this study.

#### 3.1.2 Materials

The same materials were used as in Study 1.

#### 3.1.3 Design and Procedure

This study followed a double‐blinded, within‐participant design, whereby each participant completed a personalized session and a sham session in a counterbalanced order. The stimulation parameters for the personalized session were determined using the pBO algorithm developed in Study 1. For the sham session, stimulation involved a 30 s ramp‐up and 30 s ramp‐down period, but no real stimulation was delivered afterward. Participants followed the same procedure as in Study 1.

### 3.2 Results

We planned to use a sample of 71 participants to assess the pBO‐generated tACS parameters compared to sham (placebo) tACS. This sample size was estimated based on power calculations expecting Cohen's d = 0.3, α = .05, 1−*β* = 80% in a within‐participants design. Data collection ceased at 38 participants under the preregistered sequential Bayesian analysis (*BF_01_* = 2.05; anecdotal support for the null hypothesis). This indicated that further data collection was unnecessary, as the personalized intervention did not appear to be significantly more effective compared to the sham condition (see Supplementary Figure 3). Descriptive statistics for trait questionnaires are provided in Supplementary Table 1, whereas those for state measures recorded before and after each session are provided by condition in Supplementary Table 2. The parameter combinations and their sampling frequency can be seen in Supplementary Figure 4.

#### 3.2.1 Primary Outcome: Affective Rumination

A 2 × 2 repeated measures ANOVA was conducted to examine the effects of time (pre‐ vs. post‐stimulation) and condition (personalized vs. sham) on rumination. The analysis revealed a significant main effect of time, *F*(1, 74) = 24.02, *p* < 0.001, partial *η^2^* = 0.245, with rumination scores significantly decreasing from pre‐ (*M* = 18.2, *SD* = 4.16) to post‐stimulation (*M* = 17.2, *SD* = 4.01). There was no significant main effect of condition, *F*(1, 74) = 0.05, *p* = 0.83, partial *η^2^* = 0.001. The interaction effect between time and condition was also not statistically significant, *F*(1, 74) = 2.26, *p* = 0.14, partial *η^2^* = 0.030. Thus, both conditions were followed by small reductions in rumination, but personalized tACS was not superior to sham.

#### 3.2.2 Secondary Outcomes: Sleepiness and Sleep Quality

Another 2 × 2 repeated measures ANOVA was conducted to examine the effects of time (pre‐ vs. post‐stimulation) and condition (personalized vs. sham) on sleepiness. There were no significant main effects of time and condition, nor an interaction effect (all *Fs* < 2.58, all *p*s > 0.11, all partial *η^2^s* < 0.034). Similarly, for sleep efficiency, none of the main effects or interaction effects were significant, all *F*s < 1.34, all *p*s > 0.25, all partial *η^2^s* < 0.020. For the fragmentation index, no significant effects were found either, all *F*s < 1.82, all *p*s > 0.18, all partial *η^2^s* < 0.026. Thus, sleepiness and actigraphy indices remained stable over time and did not differ between personalized and sham stimulation.

#### 3.2.3 Exploratory Analysis: Interaction Between Rumination Change and Current Amplitude

To assess whether reductions in rumination covary with reductions in sleepiness, and whether this relationship depends on stimulation intensity, we fitted a linear mixed‐effects model (REML, nloptwrap); formula: sleepiness change ratio∼current amplitude*rumination change ratio. The model included session weekday as random effect (formula: ∼1|weekday). The model's total explanatory power was moderate (conditional *R^2^* = .23) and the part related to the fixed effects alone (marginal *R^2^*) was of 0.17. The analysis revealed a significant main effect of current amplitude, *(β* = –0.16, 95% CI [–0.47, 0.16], *t*(32) = –2.42, *p* = 0.02), indicating that higher current amplitude was associated with a greater reduction in sleepiness. Additionally, the main effect of rumination change approached statistical significance, *β* = 0.30, 95% CI [–0.03, 0.63], *t*(32) = 1.87, *p* = 0.07.

**FIGURE 5:**
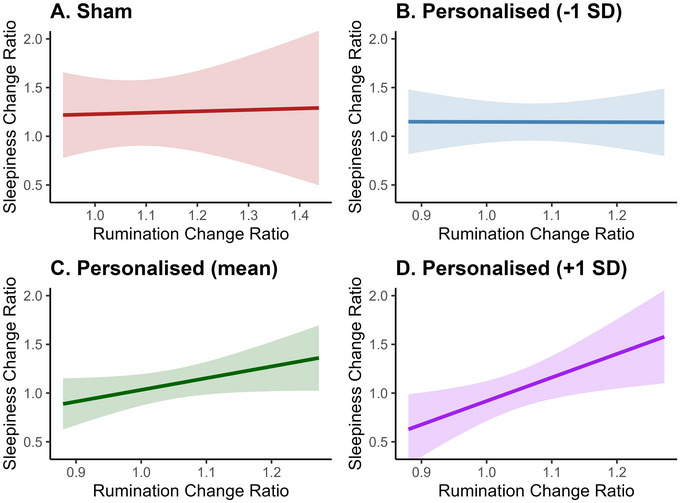
Relationship between rumination change and sleepiness change as a function of condition and current amplitude. Note: ratio values > 1 denote reductions from pre‐ to post‐stimulation, with larger values indicating a greater reduction. Panel A shows no significant association between rumination change and sleepiness change in the sham condition. Panels B‐D show simple slopes at −1 SD, mean, and +1 SD current amplitude, respectively, in the personalized condition; the coupling between larger reductions in rumination and larger reductions in sleepiness emerges only at high amplitudes. The x‐axes have different scales to accurately reflect the data range for each condition. Confidence intervals indicate uncertainty around estimates computed at the 95% level.

The interaction between rumination change and current amplitude was also significant, *β* = 0.31, 95% CI [0.04, 0.57], *t*(32) = 2.37, *p* = 0.02 (see Figure 5). Post‐hoc contrast analyses revealed that at low levels of current amplitude, rumination change was not significantly related to sleepiness change, *β* = –0.01, SE = 0.07, *t*(32.1) = –0.02, *p* = 0.98. Similarly, at moderate levels of current amplitude, rumination change was not significantly related to sleepiness change, *β* = 0.11, SE = 0.06, *t*(33.5) = 1.87, *p* = 0.07. At high levels of current amplitude, the relationship was significant, with greater reductions in rumination being associated with greater reductions in sleepiness, *β* = 0.23, SE = 0.09, *t*(33.6) = 2.60, *p* = 0.01.

Conversely, a linear mixed‐effects model controlling for the random effects of session weekday and participants had a weak total explanatory power, with a conditional *R^2^* of .09, while the proportion of variance explained by the fixed effects alone (marginal *R^2^*) was very low at < 0.001. Within the model, rumination change did not have a significant effect on sleepiness change in the sham condition, *β* = 0.02, 95% CI [–0.30, 0.35], *t*(34) = 0.15, *p* = 0.88. Therefore, higher‐amplitude stimulation was associated with lower post‐stimulation sleepiness, and the coupling between reductions in rumination and sleepiness emerged only at higher amplitudes; no such association was observed under sham.

#### 3.2.4 Side Effects

We conducted Wilcoxon signed‐rank tests to compare the severity of reported side effects (ranked from 1 = none to 4 = strong) between the sham and personalized conditions. The analysis revealed no significant differences between conditions (see Supplementary Table 3).

## 4 Discussion

The aim of the current research was to develop a novel personalized neurostimulation intervention for schoolteachers experiencing high affective work‐related rumination. First, we trained a pBO model that tailored tACS parameters based on head circumference and baseline rumination levels. We then compared the effectiveness of the personalized algorithm with a sham intervention.

In Study 1, we revealed that optimal tACS parameters for reducing rumination depend on the combination of higher current amplitude and lower frequency across head circumferences and baseline rumination levels. For individuals experiencing higher rumination on the day, as per the algorithm estimations, higher amplitudes (1.0–1.4 mA) are predicted to demonstrate the greatest effectiveness across varying head circumferences and frequency settings. Higher amplitudes, regardless of baseline rumination, have also been shown to lead to greater reductions in fragmentation index following stimulation, suggesting that tACS can improve both rumination and sleep quality. In contrast, across head circumferences and amplitudes, lower frequencies in the delta, theta, alpha, and beta bands (up to ∼25 Hz, with a peak at ∼10 Hz) appear to be most effective. However, we did not measure neurocognitive changes in this study, and reverse inference limits the strength of any definitive conclusions (Cooney et al. 2010).

In Study 2, we compared pBO‐guided tACS with sham stimulation in a preregistered double‐blinded, within‐participant design. Rumination decreased significantly following both conditions, but there was no evidence that pBO‐tACS outperformed sham on primary or secondary outcomes. A recent home‐based report of pBO‐guided tRNS improving sustained attention compared to sham (Tervo et al. 2020) demonstrates feasibility for algorithmic personalization in a different stimulation modality and domain; whether such gains extend to tACS and affective outcomes remains open. Accordingly, our preregistered null finding may constrain efficacy for the montage, parameterization, and/or affective target tested here, rather than falsifying tACS or personalization more broadly. Several non‐exclusive explanations are plausible: limited target engagement with this montage; frequency/phase misalignment; state‐dependent timing; outcome sensitivity and measurement noise; substantial between‐person variability relative to any true effect; and non‐specific or expectancy influences that compress between‐condition differences.

Alongside the condition comparison, stimulation parameter‐level analyses indicated that higher current amplitudes significantly reduced sleepiness. Additionally, greater reductions in rumination were significantly associated with greater reductions in sleepiness only at higher current amplitudes. Conversely, no association between rumination change and sleepiness change was observed in the sham condition, a pattern that closely mirrored the effects seen with lower current amplitudes in the personalized condition. This is consistent with the notion that lower current amplitudes are less likely to penetrate the scalp and effectively entrain neural oscillations (Alekseichuk et al. 2022; Johnson et al. 2020). It also suggests that in the personalized condition, changes in rumination and sleepiness were driven by a shared mechanism, with current amplitude emerging as the key factor influencing these outcomes. Therefore, the relationship between current amplitude and changes in rumination and sleepiness underscores its critical role in modulating neural activity, suggesting that higher current amplitudes may be necessary to achieve measurable cognitive and affective improvements. This also hints at a dose‐dependent response in neurostimulation, where insufficient current amplitudes may fail to elicit significant changes in oscillatory activity and behavioral outcomes (Alekseichuk et al. 2022). Nonetheless, due to the lack of tACS dose‐response studies in humans, it is unclear whether the relationship between current amplitude and response remains linear at higher current amplitudes. This could be akin to the inverted U‐shaped effects observed in tDCS and transcranial magnetic stimulation (TMS) studies concerning mental disorders (Sabé et al. 2024). Importantly, the pBO approach used in this study is not reliant on a linear relationship between current amplitude and outcomes, and can, in principle, accommodate non‐linear relationships. This adaptability ensures that current optimization is robust to individual variations and complex response patterns.

While pBO‐tACS efficacy did not exceed sham in terms of reducing affective work‐related rumination, the study demonstrates the feasibility of fully remote, AI‐personalized neurostimulation. Among those who commenced stimulation, retention was 100% in both studies. In Study 1, all participants completed the planned 5–7 at‐home sessions, for a total of 399 sessions. In Study 2, all participants completed both at‐home sessions (personalized and sham), and adverse effects were minimal and comparable between conditions. Taken together, these results indicate that pBO‐guided personalization can be delivered and monitored entirely remotely, with participants completing treatment from home rather than attending the laboratory. This finding supports the safety and practical viability of home‐based tES protocols when implemented in accordance with established guidelines, including structured screening, participant training, and adverse‐event monitoring (Fried et al. 2021; Antal et al. 2025; Nejati et al. 2025).

### 4.1 Limitations and Future Directions

Work‐related rumination is influenced by diverse occupational and individual antecedents, and the present study was not designed to adjudicate among these causal contributors. Accordingly, we treated work‐related rumination as a measurable, intervention‐sensitive cognitive process. Future studies should examine whether the current findings replicate across cohorts characterized by different profiles of key contributors to work‐related rumination. Furthermore, extending evaluation to objective, policy‐relevant endpoints would help establish occupational and public health impact across occupational contexts with high cognitive–affective demands.

Furthermore, our fully home‐based design, chosen to test teachers on working days and meet sample/scheduling demands, meant we did not record concurrent neural activity, limiting direct inferences about target engagement. In this context, personalization was based on only two features (a head anatomy proxy in the form of head circumference, together with baseline rumination), which may be too coarse for a multi‐determinant phenotype and may map only weakly onto the oscillatory processes that would need to be engaged through neurostimulation. Importantly, prior EEG studies indicate that tES can induce frequency‐dependent reconfiguration of large‐scale brain networks, including transient increases in small‐world properties and functional integration (Vecchio et al. 2018; Miraglia et al. 2022). Without concurrent EEG, it was not possible to determine whether greater reductions in rumination at specific frequencies reflected more effective entrainment, altered network efficiency, or differential sensitivity of fronto‐limbic circuits relevant to rumination.

Study 2 also delivered a single personalized tACS session; for an entrenched phenomenon such as affective work‐related rumination, a single exposure to neurostimulation might be unlikely to produce durable change. Moreover, the montage prioritized simplicity for home use over focality, potentially compromising effective current delivery to target regions. Additionally, the present study did not include an active control condition involving stimulation of a non‐target cortical site. While sham stimulation provides a robust control for placebo effects and participant expectations, the absence of a site‐specific control limits strong inferences regarding anatomical specificity of the observed effects. This design choice reflected practical and burden‐related constraints inherent to a fully home‐based, longitudinal protocol.

The above constraints are tractable. Future studies can (1) verify tACS neural effects in controlled lab sessions using electroencephalography (EEG) to quantify frequency‐ and phase‐specific engagement and to calibrate participant‐specific parameters; (2) sharpen personalization by adding low‐burden, mechanism‐relevant inputs (e.g., circadian phase/time‐of‐day, recent sleep load) and, where feasible, physiological markers, albeit at the cost of more sessions and participants for algorithm development; (3) evaluate multi‐session dosing to test the size and durability of the stimulation effects; and (4) incorporate active control sites in controlled laboratory or hybrid settings to directly test regional specificity and underlying neural mechanisms. Furthermore, bifocal or high‐definition tACS using ring configurations of small disc electrodes may enhance both behavioral and EEG outcomes by producing more targeted stimulation (Pan et al. 2023; Kuo et al. 2013). Where the home‐based setting is critical in terms of population and feasibility, more complex tACS configurations should be combined with usability aids (preconfigured caps, guided placement, remote impedance checks) to preserve feasibility.

Moreover, the home‐based design limited our ability to control participants’ activities and environments following stimulation, which could have influenced sleep outcomes and introduced variability into the results. While we opted not to include sleep diaries to avoid further complicating the home‐based setup, their use could have helped confirm the reliability of the actigraphy data. Additionally, the inability to monitor actigraphy data in real‐time posed a risk of data loss, as a few participants either forgot to wear the device or experienced issues with it falling off during the night. Notwithstanding these constraints, the at‐home approach enabled inclusive participation during working days and enhanced ecological validity; with the refinements above, it offers a scalable path for rigorous, real‐world evaluation of AI‐driven personalized neurostimulation.

## 5 Conclusions

We introduced a novel personalized home‐based neurostimulation intervention, tailored to schoolteachers experiencing heightened affective work‐related rumination. Through the development and application of a pBO model, we identified optimal tACS parameters based on individual head circumference and baseline rumination levels. On high rumination days, tACS was most effectively applied at low frequencies across head circumferences and amplitudes, and at high amplitudes across head circumferences and frequencies. High amplitudes also resulted in significantly reduced sleep fragmentation following stimulation. While both sham and personalized tACS reduced rumination, no significant differences were found between conditions. However, higher amplitudes decreased daytime sleepiness directly as well as by amplifying the effect of rumination reductions. Across both studies, feasibility was strongly supported: participants completed all sessions at home, retention was complete, and side effects were minimal and comparable to sham. Taken together, these findings suggest that remote, AI‐driven personalization of neurostimulation is practical and well tolerated, while emphasizing the need for future work to examine the effects of repeated sessions and explore advanced configurations.

## Author Contributions

**Delia Ciobotaru**: conceptualization, formal analysis, funding acquisition, project administration, investigation, visualization, writing – original draft, writing – review and editing. **Vu Nguyen**: formal analysis, project administration, software, visualization, writing – review and editing. **Alejandro Pérez**: formal analysis, investigation, project administration, writing – review and editing. **Zoe E. Clothier**: formal analysis, investigation, project administration, writing – review and editing. I**nes R. Violante**: formal analysis, supervision, writing – review and editing. **Mark Cropley**: conceptualization, formal analysis, funding acquisition, project administration, supervision, writing – review and editing. **Roi Cohen Kadosh**: conceptualization, formal analysis, funding acquisition, project administration, supervision, writing – review and editing.

## Funding

This study was funded by Joy Ventures Ltd. as part of the project “Reducing work‐related rumination through personalised neurostimulation”, with the grant awarded to the University of Surrey and Cognite Neurotechnology Ltd.

## Ethics Statement

The protocol was approved by the University of Surrey Faculty of Health and Medical Sciences Research Ethics Committee (FHMS 21–22 218 EGA). All procedures complied with the Declaration of Helsinki, and written informed consent was obtained from all participants.

## Conflicts of Interest

R.C.K. serves on the scientific advisory boards of Neuroelectrics Inc. and Tech InnoSphere Engineering Ltd. R.C.K. and V.N. filed a patent which is managed by the University of Surrey for “method for obtaining personalized parameters for transcranial stimulation, transcranial system, method of applying transcranial stimulation” (International Application No: PCT/GB2021/050123). The work in Study 1 is based on this patent. R.C.K. is a founder, director, and shareholder of Cognite Neurotechnology Ltd. D.C., A.P., Z.E.C., I.R.V., and M.C. declare no competing interests.

## Supporting information




**Supplementary Materials**: brb371264‐sup‐0001‐SuppMat.docx

## Data Availability

The data that support the findings of this study are openly available in Open Science Framework at https://osf.io/ctz4d/, reference number ctz4d.
