# Supplementary material for: Feasibility and Effectiveness of Personalized Home‐Based Neurostimulation for Teachers Experiencing Work‐Related Rumination
Source: Brain Behav. 2026 Feb 24;16(2):e71264. doi: 10.1002/brb3.71264 (PMC12931492; doi:10.1002/brb3.71264)
Supplement: Supplementary file 1 — Supplementary Materials: brb371264‐sup‐0001‐SuppMat.docx [file BRB3-16-e71264-s001.docx]

**Supplementary Figure 1**

*Frontal Brain View of Electric Field Modelling of 1mA tACS Current Amplitude at F3 and CZ*

**
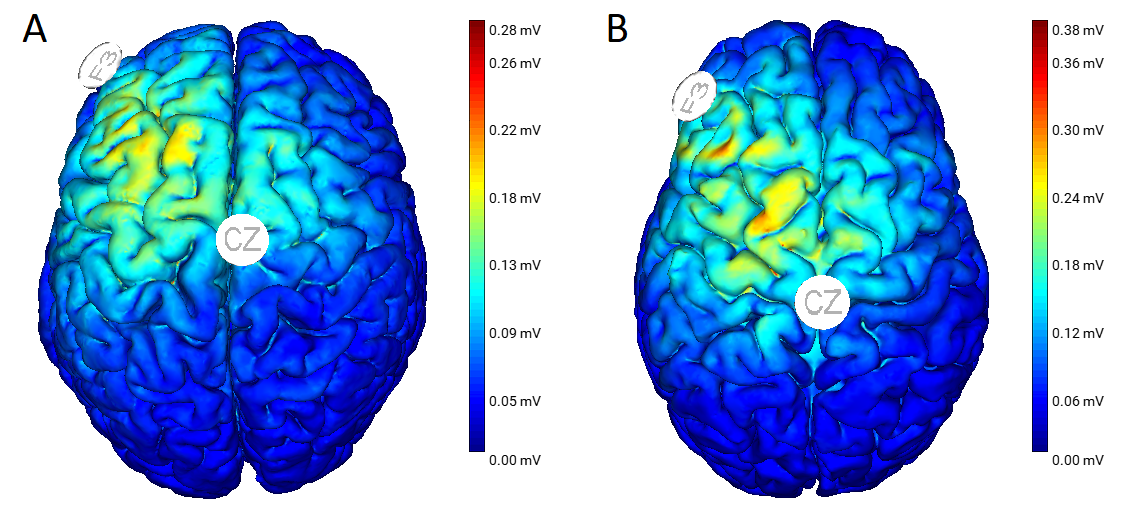
**

*Note.* Panel A shows results in a head model with a circumference of 61.9cm. Panel B shows results in a head model with a circumference of 55.4cm. The modelling was performed using the NIC2 software (v2.1.3.1.; Neuroelectrics, Barcelona, Spain).

**Supplementary Figure 2**

*Frequency and Evolution of Parameter Sampling Across Sessions in Study 1*


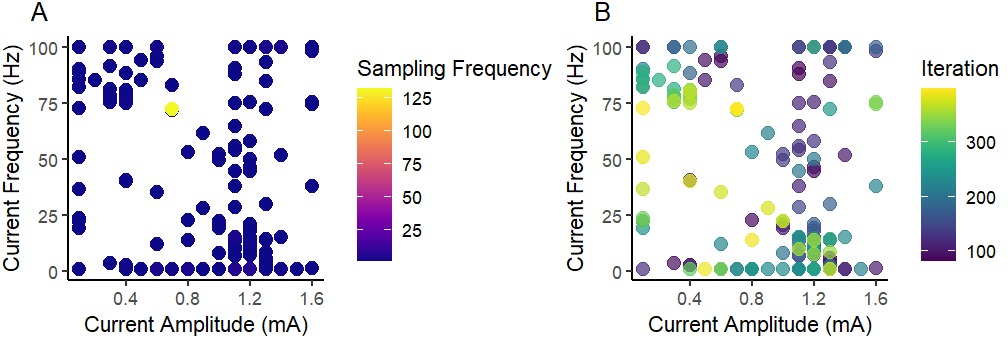


*Note.* Panel A illustrates how often different combinations of Amplitude and Frequency were sampled. Panel B indicates the session in which each combination was last explored, providing insight into the evolution of the sampling algorithm.

**Supplementary Figure 3**

*Bayesian Sequential Analysis of Affective Work-Related Rumination Change in Study 2*
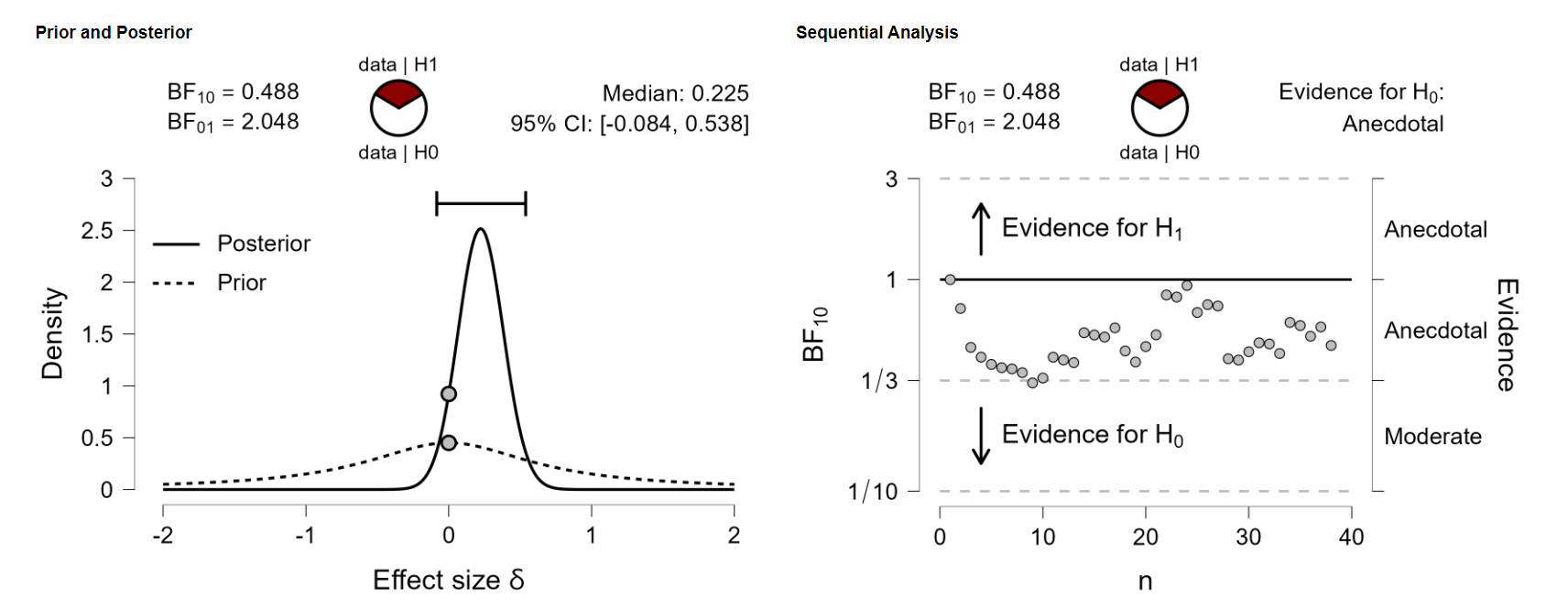


*Note.* The figure illustrates anecdotal evidence for the null hypothesis, as evidenced by a BF_01_ of 2.05.

**Supplementary Figure 4**

*Frequency of Parameter Sampling in Personalised Stimulation Sessions in Study 2*


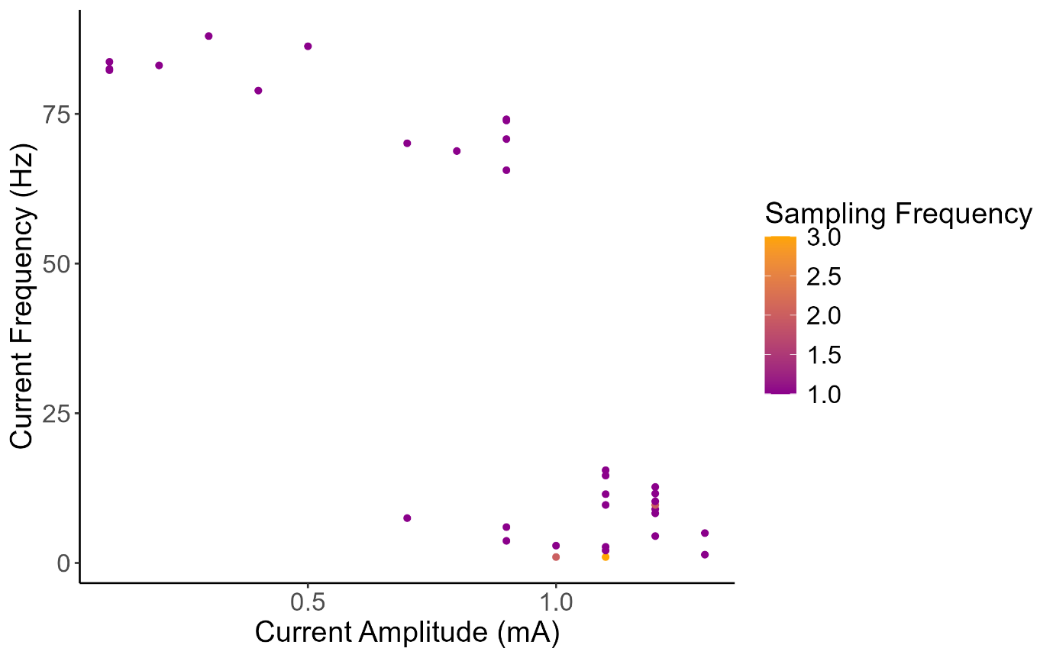


*Note.* This figure illustrates the sampling spread and frequency of different combinations of amplitude and frequency for the personalised stimulation sessions (one per participant).

**Supplementary Table 1**

*Descriptive Statistics for Trait Questionnaires Across Studies*

|  | | *Study 1* | | *Study 2* | |  |
| --- | --- | --- | --- | --- | --- | --- |
|  | | *Mean* | *SD* | *Mean* | *SD* |  |
| GAD-7 |  | 8.466 | 4.045 | 8.289 | 4.759 |  |
| PHQ-8 |  | 8.422 | 4.275 | 7.789 | 4.697 |  |
| CFQ Forgetfulness |  | 17.75 | 5.183 | 17.053 | 6.341 |  |
| CFQ Distractibility |  | 16.03 | 4.925 | 15.447 | 5.593 |  |
| CFQ False Triggering |  | 13.121 | 5.716 | 11.921 | 6.135 |  |
| PSQI |  | 7.293 | 2.894 | 7.737 | 3.210 |  |

*Note*. SD refers to Standard Deviation

**Supplementary Table 2**

*Descriptive Statistics for State Questionnaires Before and After Study 2 Sessions by Condition*

|  | Sham | | | | Personalised | | | |
| --- | --- | --- | --- | --- | --- | --- | --- | --- |
|  | Pre | | Post | | Pre | | Post | |
|  | *Mean* | *SD* | *Mean* | *SD* | *Mean* | *SD* | *Mean* | *SD* |
| Rumination | 18.3 | 4.1 | 17.0 | 3.8 | 18.2 | 4.3 | 17.5 | 4.3 |
| Sleepiness | 5.0 | 1.9 | 4.6 | 1.9 | 4.7 | 2.1 | 4.5 | 1.8 |
| Sleep Efficiency | 84.9 | 7.8 | 86.0 | 5.5 | 84.6 | 6.0 | 84.0 | 7.6 |
| Fragmentation Index | 25.2 | 11.8 | 24.9 | 10.2 | 28.2 | 12.4 | 27.5 | 11.1 |

*Note*. SD refers to Standard Deviation

**Supplementary Table 3**

*Wilcoxon Signed-Rank Test Comparison of Side Effects Between Conditions in Study 2*

|  | Sham | | | Personalised | | Wilcoxon Test | | |
| --- | --- | --- | --- | --- | --- | --- | --- | --- |
|  | *Mean* | *SD* | *Mean* | | *SD* | *z* | *p* | |
| Itching | 1.579 | 0.599 | 1.474 | | 0.557 | 0.827 | | 0.359 |
| Skin Redness | 1.053 | 0.226 | 1 | | 0 | NaN | | NaN |
| Headache | 1.237 | 0.431 | 1.132 | | 0.414 | 1.26 | | 0.182 |
| Scalp Pain | 1.053 | 0.226 | 1.079 | | 0.273 | -0.535 | | 0.773 |
| Burning Sensation | 1.053 | 0.226 | 1.053 | | 0.226 | 0 | | 1 |
| Metallic Taste | 1.079 | 0.273 | 1.053 | | 0.226 | 0.535 | | 0.773 |
| Sleepiness | 1 | 0 | 1.079 | | 0.273 | NaN | | NaN |
| Trouble Concentrating | 1.368 | 0.589 | 1.237 | | 0.542 | 1.185 | | 0.235 |
| Nervousness | 1.105 | 0.311 | 1.053 | | 0.226 | 0.913 | | 0.424 |
| Discomfort | 1.105 | 0.311 | 1.026 | | 0.162 | 1.604 | | 0.149 |
| Unpleasant Sensation | 1.158 | 0.37 | 1.079 | | 0.273 | 1.214 | | 0.233 |
| Dizziness | 1.026 | 0.162 | 1.053 | | 0.226 | -1 | | 1 |
| Nausea | 1.079 | 0.359 | 1.105 | | 0.509 | 0 | | 1 |
| Visual Sensation | 1.026 | 0.162 | 1 | | 0 | NaN | | NaN |

*Note*. SD refers to Standard Deviation. NaN refers to results that are not a number, in this case due to the lack of variance in measures for one of the conditions.
